# Biosynthesis of 2-hydroxyisobutyric acid (2-HIBA) from renewable carbon

**DOI:** 10.1186/1475-2859-9-13

**Published:** 2010-02-25

**Authors:** Thore Rohwerder, Roland H Müller

**Affiliations:** 1 Helmholtz Centre for Environmental Research, Department Environmental Microbiology, Permoserstr. 15, 04318 Leipzig, Germany

## Abstract

Nowadays a growing demand for green chemicals and cleantech solutions is motivating the industry to strive for biobased building blocks. We have identified the tertiary carbon atom-containing 2-hydroxyisobutyric acid (2-HIBA) as an interesting building block for polymer synthesis. Starting from this carboxylic acid, practically all compounds possessing the isobutane structure are accessible by simple chemical conversions, e. g. the commodity methacrylic acid as well as isobutylene glycol and oxide. During recent years, biotechnological routes to 2-HIBA acid have been proposed and significant progress in elucidating the underlying biochemistry has been made. Besides biohydrolysis and biooxidation, now a bioisomerization reaction can be employed, converting the common metabolite 3-hydroxybutyric acid to 2-HIBA by a novel cobalamin-dependent CoA-carbonyl mutase. The latter reaction has recently been discovered in the course of elucidating the degradation pathway of the groundwater pollutant methyl *tert*-butyl ether (MTBE) in the new bacterial species *Aquincola tertiaricarbonis*. This discovery opens the ground for developing a completely biotechnological process for producing 2-HIBA. The mutase enzyme has to be active in a suitable biological system producing 3-hydroxybutyryl-CoA, which is the precursor of the well-known bacterial bioplastic polyhydroxybutyrate (PHB). This connection to the PHB metabolism is a great advantage as its underlying biochemistry and physiology is well understood and can easily be adopted towards producing 2-HIBA. This review highlights the potential of these discoveries for a large-scale 2-HIBA biosynthesis from renewable carbon, replacing conventional chemistry as synthesis route and petrochemicals as carbon source.

## Background

### Building-block chemical 2-HIBA

**Figure 1:**
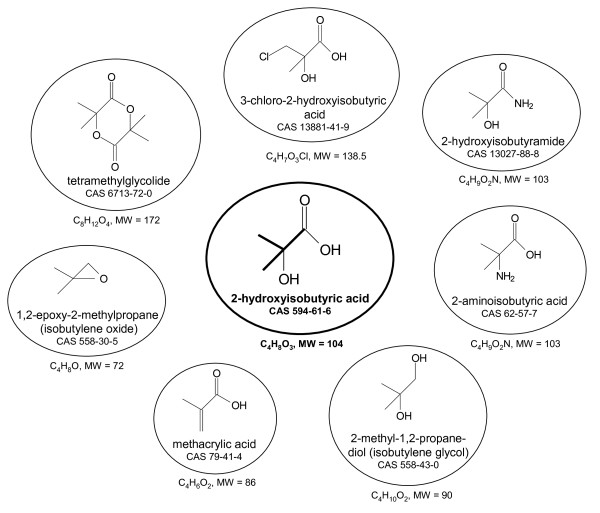
**Various substances that can easily be derived from 2-HIBA by standard chemical conversions**.

In the future, the feedstock for the chemical industry will not any longer be delivered from petroleum but will depend on renewable biomass. This does not mean that materials currently produced by the industry will be replaced by totally new ones. However, synthesis routes of the chemical industry often differ from the pathways that are found in biology. While chemistry produces polymers and other materials origin mainly from petroleum-based hydrocarbons, biological systems normally feed on carbohydrates, alcohols or other renewable carbon which are already at a highly oxidized state. Accordingly, the central intermediates from which all other compounds are synthesized, the building-block chemicals, are not the same in chemical and biological systems. However, this situation will change when the resource base for chemical production is shifted from fossil feedstock to renewable carbon [1,2]. A good example is 2-hydroxyisobutyric acid (2-HIBA) whose chemistry and biochemistry also differ significantly. This tertiary carbon atom-containing C4 carboxylic acid is currently not a high-volume product of the chemical industry but only a specialty chemical. It is used to some extend as a pharmaceutical intermediate and also as a complex-forming agent for lanthanide and actinide heavy metals [3]. In the future, however, it could be used as a building-block chemical for many polymer precursors bearing the isobutane carbon skeleton (Figure 1). All these chemicals are mainly used for polymer production. Above all, the methyl ester of methacrylic acid, which can be synthesized by dehydration of 2-HIBA or via the corresponding amide, is polymerized to polymethylmethacrylate (PMMA) for the production of acrylic glass, durable coatings and inks [4,5]. For this compound alone, the market exceeds 3 million tons. Other branched C4 carboxylic acids, e. g. chloro and amino derivatives of 2-HIBA [6-9], as well as isobutylene glycol and its oxide are also used in polymers and for many other applications [10-13]. In addition, by analogy with lactide from lactic acid, the corresponding dimer of 2-HIBA, tetramethylglycolide, can easily be formed for further polymerization [14,15].

### Lack of a suitable metabolism

Currently, polymer precursors having the isobutane structure are synthesized from petroleum-derived C2 to C4 hydrocarbons, such as ethylene, acetone, isobutane and isobutylene, but not from renewable biomass. Biology, on the other hand, prefers to synthesize carboxylic acid from biobased carbon rather than to reduce it to hydrocarbons. In line with this, it has been predicted that several organic acids, such as 3-hydroxypropionic and succinic acid, will be important building-block chemicals when industrial production will shift from petroleum-based chemistry to biotechnology from renewable carbon [1,16]. Although large-scale production of the organic acid building-blocks has not yet been established, their high market potential has fueled significant efforts to do so. In most cases, the underlying biochemistry is well understood and has been known for decades. In contrast, research on the conversion or synthesis of 2-HIBA by microorganisms or other biological systems has not attracted much attention as it is rarely found in nature and is not part of the main metabolic pathways. Obviously due to the lack of a suitable metabolism, a biobased 2-HIBA has not been predicted to be an important building-block organic acid [1], although it is known that many high-volume polymer chemicals can easily be synthesized from it (Figure 1). Until recently, only the biohydrolysis of acetone cyanohydrin as a partially biotechnological route to 2-HIBA has been proposed [17], but convincing approaches depending on renewable carbon were not available.

### MTBE: driver for the evolution of novel enzymes?

In the last years, significant progress on elucidating 2-HIBA metabolism has been made when research focused on the environmental fate of the fuel oxygenate methyl *tert*-butyl ether (MTBE) since 2-HIBA has been identified as an intermediate in its biodegradation pathway [18]. MTBE is an ether compound formally derived from the alcohols methanol and *tert*-butanol. In practice, it is produced by reacting methanol with isobutylene. Hence, the ether bears a *tert*-butyl residue that belongs to the group of chemicals with an isobutane-like structure (Figure 1). MTBE is used to some extent as a cheap organic solvent. However, its rise to a mass product of the chemical industry started in the late 1970s as a gasoline additive [19]. At the beginning, only small amounts, comprising less than 1% of the gasoline, were added for raising the octane number. Then, its use steadily increased to about 5 to 15% as a so-called fuel oxygenate for optimizing combustion. Today, the market for MTBE and related fuel oxygenates exceeds 20 million tons. While these compounds help to reduce CO emission, they also tend to easily pollute groundwater when gasoline is spilled or leaked at gas stations. MTBE turned out to be quite recalcitrant against biodegradation, likely due to the xenobiotic character of the *tert*-butyl residue [20].

**Figure 2:**
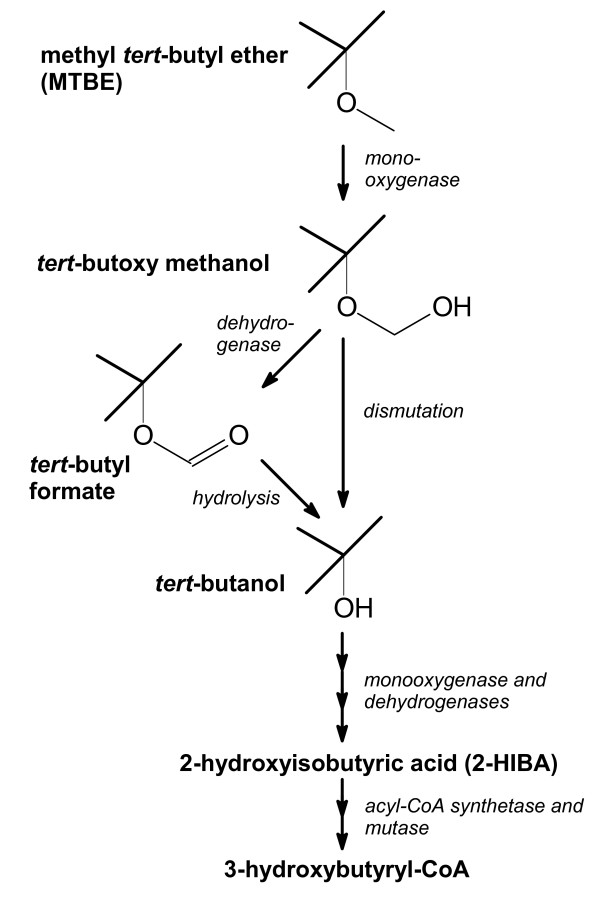
**Pathway of aerobic MTBE degradation**. In bacteria, MTBE and also other ethers used as fuel oxygenates are initially attacked by various monooxygenases [20]. The resulting *tert*-butoxy methanol may spontaneously dismutate to *tert*-butanol and formaldehyde or is further oxidized to *tert*-butyl formate. The latter ester can be hydrolyzed to *tert*-butanol and formic acid. A second monooxygenase catalyzes the hydroxylation of *tert*-butanol to 2-methyl-1,2-propanediol which is oxidized via the corresponding aldehyde to 2-HIBA. The latter is then isomerized to the common metabolite 3-hydroxybutyryl-CoA [39]. For further details see also Figure 3b and c.

Now, after more then a decade of intensive research, a few bacterial strains are known capable of growing on MTBE as sole source of carbon and energy [20,21]. Probably, the massive use and the resulting widespread groundwater pollution has been the driver for the evolution of bacterial strains possessing a suite of special enzymes which enable MTBE mineralization (Figure 2). After several oxidation steps, 2-HIBA as the last MTBE intermediate carrying the isobutane skeleton is infiltrated into the common metabolism by a mutase catalyzing the conversion to the unbranched 3-hydroxybutyric acid. As the involved enzymes seem to be adapted to the *tert*-butyl residue, they may be of biotechnological use for the interconversion of chemicals having the isobutane structure or a similar one (Figure 1). The mutase stands out against all other MTBE-related enzymes as it connects the "common" world of biobased compounds with the special structures derived from 2-HIBA.

**Figure 3:**
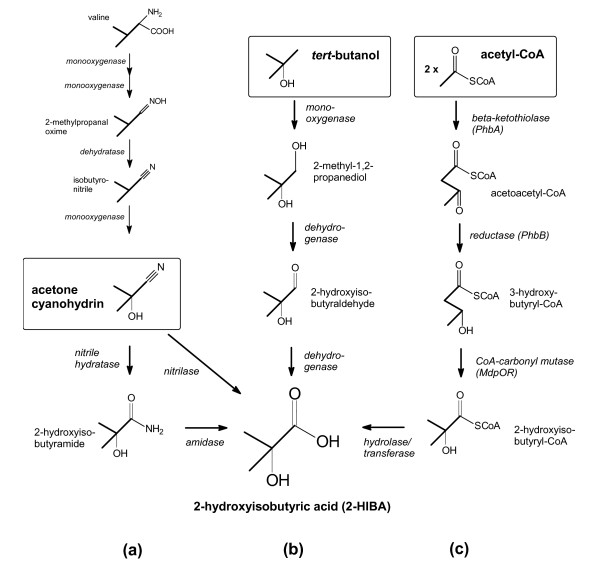
**Proposed biotechnological routes to 2-HIBA**. (a) Biohydrolysis of acetone cyanohydrin, (b) biooxidation of *tert*-butanol and (c) bioisomerization of 3-hydroxybutyryl-CoA that is synthesized from acetyl-CoA. The known biosynthesis sequence of the linamarin pathway leading from valine to acetone cyanohydrin is also indicated.

In the course of these research activities, significant progress on the biochemistry of 2-HIBA has been achieved. Besides biohydrolysis, now other pathways such as biooxidation processes and, above all, a novel bioisomerisation reaction can be employed for producing 2-HIBA. These findings prepare for the first time the ground for biotechnological routes producing 2-HIBA from renewable organic compounds, such as carbohydrates, alcohols or fatty acids. Here, we describe and discuss the old and new biotechnological pathways to 2-HIBA in detail. Furthermore, the potential of these synthesis routes for a large-scale biosynthesis of 2-HIBA from renewable carbon is outlined.

## Possible biotechnological routes

### Biohydrolysis of acetone cyanohydrin

Since the late 1980s, processes for the biohydrolysis of aliphatic 2-hydroxynitriles to 2-HIBA and other 2-hydroxy carboxylic acids have been developed [17,22,23]. Nitrile-hydrolyzing enzymes are widespread in nature and are employed by bacteria and fungi for degradation of biogenic nitriles, which are mainly of plant origin [24]. In principle, two different pathways are available (Figure 3a). On the one hand, the nitrile can be directly hydrolyzed by a nitrilase to the carboxylic acid. On the other hand, the same result can be obtained by combining activities of suitable nitrile hydratases and amidases. In this two-step reaction sequence, the corresponding amide is formed as an intermediate and then hydrolyzed to the carboxylic acid. Whereas nitrile hydratases for aliphatic nitriles have been known for many years, nitrilases for the same substrates were not available until the 1990s [25,26].

For producing 2-HIBA, nitrile-hydrolyzing activities from strains of various genera, such as *Acidovorax*, *Comamonas*, *Pseudomonas *and *Penicillium*, have been tested [17,22,23]. In principle, whole cells or enzyme preparations have to be incubated with acetone cyanohydrin in aqueous solution where 2-HIBA is formed. A severe disadvantage of the process is the instability of acetone cyanohydrin at the near-neutral pH value required for an efficient nitrile-hydrolyzing enzyme activity. The decomposition of acetone cyanohydrin in the reaction mixture results in the accumulation of acetone and cyanide which inhibit the enzyme activity. Consistently, some effort has been made for screening for cyanide-resistant nitrile-hydrolyzing enzymes [27]. As many relevant enzymes are well characterized, optimization may also be accomplished by *in vitro *mutagenesis approaches. Recently, for example, protein engineering was used for increasing specific activity of a nitrilase from *Acidovorax facilis *72W for the synthesis of 3-hydroxy carboxylic acids [28].

Although an efficient acetone cyanohydrin-hydrolyzing enzymatic system could be developed in the future, it has to be stressed that this route is only partially based on biotechnology as the nitrile will obviously be synthesized by petrochemical processes. Theoretically, a biosynthesis route to acetone cyanohydrin might be derived from the pathway of linamarin production found in plants [29,30]. The latter compound belongs to the group of cyanogenic glucosides which are a kind of chemical defense response. In linamarin, the hydroxyl group of acetone cyanohydrin forms a beta-glucosidic bond with glucose. When plant tissue is disrupted by chewing insects, beta-glucosidases and alpha-hydroxynitrilases get access to the glucoside and toxic hydrogen cyanide and acetone are released. Before glucosidation to form linamarin, acetone cyanohydrin is synthesized in a multi-step process from the amino acid valine via the corresponding oxime (Figure 3a). In the last years, there has been significant progress in elucidating this pathway and most of the involved enzymes are identified [29]. In this connection, also the *in vitro *reversibility of the hydroxynitrile lyase reaction has to be mentioned [31]. Thus, acetone cyanohydrin could also be enzymatically produced from acetone and hydrocyanic acid. However, a large-scale biotechnological route to acetone cyanohydrin is not realistic due to the instability and toxicity of this compound.

### Biooxidation of tert-butanol

A biooxidation route to 2-HIBA (Figure 3b) has recently been derived from the bacterial degradation pathway of MTBE via *tert*-butanol (Figure 2). The responsible enzymes and encoding genes from the MTBE-degrading bacterium *Mycobacterium austroafricanum *IFP 2012 have been characterized [20]. In this strain, a none-heme alkane monooxygenase is hydroxylating *tert*-butanol. The resulting diol is further oxidized by two novel dehydrogenases, designated as MpdB and MpdC [20], to the carboxylic acid. The production of 2-HIBA employing the mycobacterial enzymes has been patented [32]. More recently, the enzyme system for the oxidation of *tert*-butanol has also been described for the MTBE-degrading bacterial strains *Methylibium petroleiphilum *PM1 [33] and *Aquincola tertiaricarbonis *L108 [34]. In both strains, not an alkane monooxygenase but a phthalate dioxygenase-like enzyme catalyzes the hydroxylation of *tert*-butanol. This enzyme belongs to the family of mononuclear iron Rieske oxygenases [35]. So far, *A. tertiaricarbonis *L108 is the most efficient MTBE-degrader among the known bacterial strains showing autarkic growth on this fuel oxygenate [21]. Highly likely, this outstanding degradation potential is rooted in a set of specialized enzymes well adapted for degrading MTBE and its metabolites. Hence, employing the relevant enzymes found in strain L108 may be the most promising approach for producing 2-HIBA from *tert*-butanol [36].

Biooxidation of *tert*-butanol may be an interesting route for producing 2-HIBA. Similar to the approaches employing nitrile-hydrolizing enzymes and starting at acetone cyanohydrin, the substrate of the biological steps has to be synthesized by petrochemical routes. *tert*-Butanol is a bulk chemical and widely used as solvent and intermediate in chemical syntheses [37]. It is also subjected to chemical oxidation for producing methacrylic acid [4]. The now available enzymatic route would be highly selective and less by-products would be formed compared to the chemical oxidation process. Most attractive for producing 2-HIBA, however, would be a biotechnological process which is able to use substrates from renewable resources.

### Bioisomerization of 3-hydroxybutyric acid

A biobased route to 2-HIBA is indeed possible, namely by employing the second relevant metabolic sequence derived from the degradation pathway of MTBE, the isomerisation of 3-hydroxybutyric acid (Figure 3c). Contrary to the biohydrolysis and biooxidation processes for establishing a biotechnological pathway to 2-HIBA, the bioisomerisation approach does not use a substrate unknown to many biological systems but a common metabolite. 3-Hydroxybutyric acid can easily be synthesized from renewable carbon by numerous microorganisms. Thus, the bioisomerisation proposal is the only process currently available for a completely biotechnological production of 2-HIBA [38].

**Figure 4:**
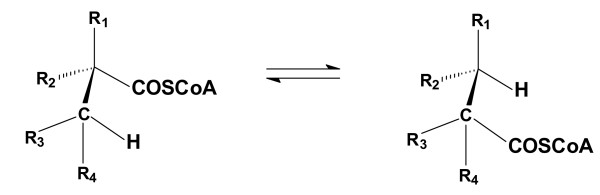
**Carbon skeleton rearrangement catalyzed by cobalamin-dependent CoA-carbonyl mutases**. Conversions for carboxylic acids having residues R1 to R4 out of H, CH_3_, OH and COOH have already been described.

Search for the enzymatic step connecting the MTBE intermediate 2-HIBA to the general assimilating and dissimilating carbon metabolism led to the discovery of a novel mutase in the bacterial strain *A. tertiaricarbonis *L108 [39]. In the bacterial MTBE degradation pathway, this enzyme catalyzes the reversible conversion of the branched 2-HIBA into the linear 3-hydroxybutyric acid, more precisely, the Coenzyme A (CoA)-activated thioesters are the substrates of the mutase. In doing this, the MTBE-specific pathway with quite unusual molecules characterized by the *tert*-butyl moiety is connected with the common metabolism, as 3-hydroxybutyryl-CoA can easily be assimilated or oxidized to carbon dioxide. The so-called 2-hydroxyisobutyryl-CoA mutase is a new representative of the CoA-carbonyl mutase family. These enzymes catalyze the 1,2-rearrangement of the CoA-activated carboxyl group in the carbon skeleton of their substrates [40,41] (Figure 4). In this quite complicated reaction, the cofactor adenosylcobalamin is used to create radical intermediates. Among these enzymes is the widespread methylmalonyl-CoA mutase (EC 5.4.99.2) catalyzing the interconversion of methylmalonyl-CoA and succinyl-CoA, which plays a role in the conversion of branched-chain amino acids, odd-chain fatty acids and cholesterol. Another mutase, the isobutyryl-CoA mutase (EC 5.4.99.13), is only known from bacteria and is responsible for the interconversion of isobutyryl-CoA and butyryl-CoA. This enzyme has been attributed to play a role in providing building blocks for synthesis of polyketide antibiotics in *Streptomyces *spp. [42,43]. More recently, a mutase has been characterized catalyzing the interconversion of ethylmalonyl-CoA and methylsuccinyl-CoA [44]. Besides these mutases, analogous rearrangements have been proposed in several bacterial pathways for the anaerobic degradation of hydrocarbons and pivalic acid [40].

As the stereospecifc 1,2-rearrangement of carboxylic acids can hardly be managed by chemical synthesis, enzymes possessing this capability have a great biotechnological potential. Not only potential commodities such as 2-HIBA (Figure 3c) but also specialty chemicals, e. g. chiral precursors for drug synthesis, may be produced with the help of the mutases in the future. A prerequisite would be a better understanding of the substrate specificity. Sequence comparison of the substrate-binding site, however, reveals high similarity among the known enzymes. This is due to the fact that only the conversion of CoA-activated substrates is catalyzed. Consequently, most of the binding site interacts with the quite large coenzyme (MW = 768) and not with the specific carboxylic acid (e. g. MW = 118 in the case of methylmalonic acid). Indeed, only a few amino acid residues have been identified to specifically interact with the carboxylic acid substrate [40,45]. On the other hand, simple replacements of these residues have not yet generated active enzymes [46].

Having a central position in the MTBE degradation pathway makes the new 2-hydroxyisobutyryl-CoA mutase an important tool in developing for the first time a completely biotechnological process for producing 2-HIBA. That is, in case of running a suitable biological system not along the MTBE degradation path, but in the opposite direction and feeding simple substrates which are metabolized via acetyl-CoA, 3-hydroxybutyryl-CoA can easily be produced by well-known enzymatic reaction sequences (Figure 3c). Now, due to the reversibility of the isomerization reaction, the mutase will take the 3-hydroxybutyryl-CoA and convert it into 2-hydroxyisobutyryl-CoA. It has to be highlighted that the enzyme accomplishes this important job very elegantly in only one step. Whole cells of the wildtype strain of *A. tertiaricarbonis *already excrete 2-HIBA into the medium when feed with 3-hydroxybutyrate in the absence of oxygen [38]. Under these conditions, the substrate cannot be dissimilated by the cells but is CoA-activated and converted into 2-hydroxyisobutyryl-CoA. The reversibility of the mutase reaction has also been proved using cell-free enzyme preparations of *A. tertiaricarbonis *[38].

**Figure 5:**
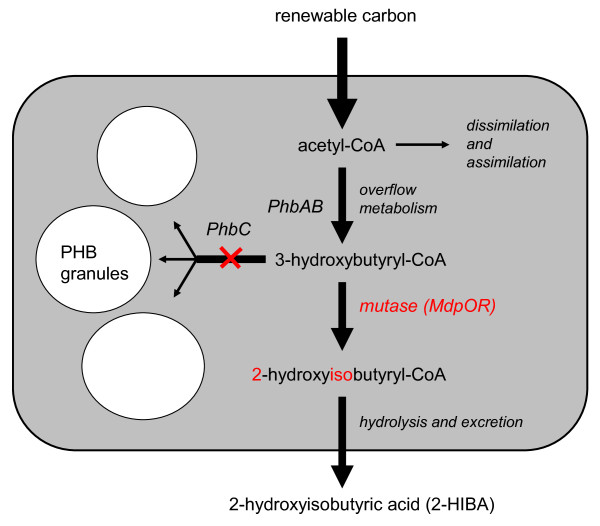
**Connection of PHB biosynthesis steps to the proposed whole-cell production of 2-HIBA employing the bioisomerization route of aerobic MTBE degradation**. In the absence of PHB polymerase activity, a biological system having the ability for overflow metabolism will accumulate 3-hydroxybutyryl-CoA from any suitable carbon source. This common metabolite will be converted to 2-hydroxyisobutyryl-CoA which is hydrolyzed and excreted.

Biological systems for producing 3-hydroxybutyryl-CoA and the free acid 3-hydroxybutyrate are well known. An endless number of bacteria synthesizes the intracellular reserve material poly-3-hydroxybutyrate (PHB) which is formed from enzymatic polymerization of 3-hydroxybutyryl-CoA [47]. In the case of 2-HIBA synthesis, similar systems can be used, e. g., by simply replacing the PHB synthase with the 2-hydroxyisobutyryl-CoA mutase. In few words, the new route employing the 2-hydroxyisobutyryl-CoA mutase could be described as a simple modification of the PHB and 3-hydroxybutyric acid biosynthesis process (Figure 5). Considering the advanced stage of development of the latter biotechnology [48,49], the new route to 2-HIBA is very promising as most of the know-how can be adopted.

## Potential for large-scale production

### Required host physiology

As already indicated above, the bioisomerization process (Figure 3c) is currently the only proposal for a biotechnological production of 2-HIBA from renewable carbon resources. Moreover, its connection to the PHB metabolism is a great advantage towards developing a large-scale biotechnological production of 2-HIBA. For many decades, the PHB pathway has been investigated for producing biodegradable plastics and much effort has been spent on studying the enzymatic steps and their regulation [47,50]. Consequently, the metabolism resulting in 3-hydroxybutyryl-CoA and PHB is well understood and the corresponding biotechnological process well established. However, as the synthesis of 2-HIBA and PHB only share part of the enzymatic steps but finally deviate when the product is formed (Figure 5), distinctive physiological features might be required from a suitable host system. Besides expressing a functional 2-hydroxyisobutyryl-CoA mutase, a high-rate production of its substrate, the 3-hydroxybutyryl-CoA, has to be achieved.

So far, the 2-hydroxyisobutyryl-CoA mutase has only been detected in bacteria [39,40]. Among them are the MTBE-degrading strains of the beta-proteobacterial genera *Methylibium *and *Aquincola*. Consequently, the genes encoding for the two mutase subunits have been designated *mdpO *and *mdpR *(*mdp *for MTBE degradation pathway) [33]. In addition, the mutase occurs in a few other bacterial strains not associated with fuel oxygenate mineralization [51]. Most of these strains also belong to the beta-proteobacteria, indicating a possible disposition of this phylogenetic group for 2-HIBA metabolism. On the other hand, the genome of the Gram-positive strain *Nocardioides *JS614 is encoding for the 2-hydroxyisobutyryl-CoA mutase and the strain is able to grow on 2-HIBA [51]. Obviously, a phylogenetic barrier for transfer and expression of the mutase genes does not exist. In line with this, expression of another CoA-carbonyl mutase, the closely related isobutyryl-CoA mutase, from the Gram-positive *Streptomyces cinnamonensis *by the gamma-proteobacterium *E. coli *has been achieved [45]. Moreover, the heterologous expression of bacterial reaction sequences involving the methylmalonyl-CoA mutase has already been established in yeast for complex polyketide biosynthesis [52].

For the bioisomerization to 2-HIBA, any biological system would be suitable as a catalyst that can channel the carbon flux to 3-hydroxybutyryl-CoA. An essential trait would be the ability for overflow metabolism. This holds equally true for the accumulation of PHB. Disrupting the latter process for connecting the carbon flow to 2-hydroxyisobutyryl-CoA mutase will finally lead to the accumulation of the building-block chemical 2-HIBA (Figure 5). Natural PHB synthesis is only found in bacteria and has mainly been studied in Gram-negative strains [47]. However, also Gram-positive strains have the potential for PHB production [53]. In addition, the PHB synthesis genes (*phbABC*) have already been introduced into yeast and other organisms [47,48]. In a 2-HIBA producing system, of course, activity of the PHB synthase (PhbC) has to be eliminated. As a countermove, the mutase genes *mdpOR *[33,39] have to be expressed. Recently, modifications for producing not the polymer PHB but the enantiopure monomers (*R*)-3-hydroxybutyric acid [54,55] or (*S*)-3-hydroxybutyric acid [56] have been developed. Thus, the combined expression of the relevant PHB synthesis and mutase genes should lead to 2-HIBA production in a large variety of hosts.

### Possible feedstocks

However, a 2-HIBA biotechnology faces the same economic problems as any other bioprocess aiming to produce a commodity chemical. Although it is likely that a robust and high yield fermentation could be established, high costs for substrates and downstream processing would be major drawbacks. Nevertheless, considering the particular features of the bioisomerization pathway makes it a very realistic scenario that a large-scale production of biobased 2-HIBA could be established in the near future. This will be outlined in the following.

Due to the fact that PHB synthesis and the bioisomerization process to 2-HIBA follow the same pathway from acetyl-CoA via acetoacetyl-CoA to 3-hydroxybutyryl-CoA (Figure 3c), practically all substrates and organisms which have already been tested for producing the polymer are suitable systems. As carbon substrates, any carbohydrates, alcohols, organic (fatty) acids, polyols etc. can be used. However, current biotechnological processes for producing fuels and chemicals are faced with rising prices as they mainly depend on simple sugars or alcohols as carbon source. Consequently, process development should focus on second generation substrates. In line with this, one of the present focuses on PHB and, more general, polyhydroxyalkanoate (PHA) production has been on lowering the production costs. In this connection, recently low-cost substrates, such as wood biomass [57], starch [58] as well as cane molasses and other agricultural feedstocks [59], have been successfully used for PHA production. With respect to the carbon conversion efficiency and productivity, the same high values for 2-HIBA can be expected as already achieved for PHA [47-49].

### Harvesting technologies

In comparison with PHA which forms intracellular granules, harvesting and processing of the target product would be easier in the case of 2-HIBA. The latter is a water-soluble monomer that is excreted into the growth medium [38]. Consequently, the producing cells need not to be disrupted prior to product separation. In the case of PHB, the released granules are typically separated by centrifugation which significantly contributes to the processing costs [47]. On the contrary, separation technologies already employed for biotechnologically produced acetic, lactic and succinic acid can be adopted as in all these cases structurally related low-weight carboxylic acids will be processed. In this connection, separation processes such as the traditional crystallization as well as solvent extraction and ion-exchange separation have been established [1,16].

## Conclusions

Although a variety of industrial relevant precursors for polymers bearing the isobutane carbon skeleton are easily accessible from the C4 compound 2-HIBA, it is not included in recent reports on potential biobased building-block chemicals. This ignorance can be explained by the lack of a feasible biotechnological route to 2-HIBA. For decades, only the inefficient biohydrolysis of acetone cyanohydrin as a partially biotechnological process has been known. More recently, biooxidation of *tert*-butanol to 2-HIBA has been described. However, more attractive for the production of 2-HIBA at industrial scale would be a pathways starting from renewable resources. Due to the discovery of a novel CoA-carbonyl mutase in MTBE-degrading bacteria, now a promising approach employing enzymes of PHB synthesis and the 2-hydroxyisobutyryl-CoA mutase can be proposed. Here, well established biotechnology is slightly modified by adding only one enzymatic step catalyzing very elegantly the 1,2-rearrangement of the common metabolite 3-hydroxybutyric acid to 2-HIBA.

## Competing interests

The authors declare that they have no competing interests.

## Authors' contributions

The authors contributed equally to this work.
